# “I’m not sure whether I will implement it”: exploring barriers and facilitators to implementing a digital “healthy eating” resource in early education and care settings - teachers’ perspectives

**DOI:** 10.1186/s12889-024-19014-7

**Published:** 2024-06-05

**Authors:** Sissel H. Helland, Kristine Vejrup, Nina C. Overby

**Affiliations:** 1 Department of Nutrition, and Public Health, Faculty of Health and Sport Sciences, University of Agder, PO Box 422, Kristiansand, 4604 Norway; 2 Norwegian Armed Forces Joint Medical Services, Institute of Military Epidemiology, Oslo, Norway

**Keywords:** Implementation, Early education and care, Intervention, Digital, Nutrition

## Abstract

**Background:**

Scaling up effective interventions to promote healthy eating habits in children in real-world settings is a pressing need. The success of implementation hinges crucially on engaging end-users and tailoring interventions to meet their specific needs. Building on our prior evaluation of a digital “healthy eating” resource for early childhood education and care (ECEC) staff; this qualitative study aims to pinpoint the barriers and facilitators that influence the successful implementation of such interventions.

**Methods:**

We conducted twelve semi-structured interviews with ECEC teachers in a Norwegian municipality. Interview participants were later invited to participate in focus groups where two discussions were conducted with five of the participants to reflect on the initial interview findings. Thematic analysis, facilitated by NVivo software, was employed to analyse the data, aiming to identify and summarize teachers’ subjective experiences and perspectives.

**Results:**

Teachers’ perceptions of barriers to the implementation of an upcoming digital “healthy eating” resource included: *(1) No established tradition of using digital resources at work; (2) Uncertainty regarding the achievable outcomes of implementation; (3) Perception of the new “healthy eating” resource as cooking-focused and unnecessary;* and *(4) Hectic everyday life serving as a barrier to the long-term use of a digital resource.* Facilitators for implementation included: *(1) A user-friendly format; (2) Newsletters featuring seasonal tips inspire and serve as effective reminders; (3) Emphasis on research and legislation;* and *(4) Structuring the resource as a series and an idea bank.*

**Conclusions:**

The findings underscore the essential need for tailored strategies and comprehensive support structures to successfully implement a culturally appropriate digital “healthy eating” resource for ECEC staff, ensuring effectiveness and feasibility.

**Trial registration:**

This study was not registered in a trial registry as it is not a clinical trial or intervention study but serves as a pilot for the *Nutrition Now* study, trial identifier ISRCTN10694967 (10.1186/ISRCTN10694967), registration date: 19/06/2022.

**Supplementary Information:**

The online version contains supplementary material available at 10.1186/s12889-024-19014-7.

## Background

Implementing “healthy eating” interventions in early childhood and care (ECEC) settings (e.g. kindergartens, nursery school, preschools, daycare, childcare services), may positively impact the diets of preschoolers [1–3]. As dietary behaviors developed during early childhood are known to track into adulthood [4], there is a need to scale-up and implement effective interventions promoting healthy eating habits among children in real-world settings [2, 5]. The implementation of evidence-based interventions is a multifaceted and gradual process, not without challenges [6–8].

Active engagement of users and qualitative research, as emphasized by Gittelsohn et al. (2006), are crucial for gaining insights into user perspectives [9]. Conducting qualitative research that considers the local context and acknowledges implementation barriers and facilitators is essential for a deeper understanding of human behavior affecting implementation success [1]. For instance, such exploration helps assess the correspondence between community needs and evidence-based interventions, aiding in program adoption decisions [6]. Building on this, a systematic review from 2020 on implementation support strategies in ECEC settings indicates that comprehensive approaches addressing multiple barriers are most effective in promoting evidence-based nutrition interventions and improving children’s dietary intake [8].

According to Fixsen et al. (2005), achieving success in implementation requires identifying critical steps in the process and understanding the key factors shaping their outcomes. They have found that the following stages are important in the process of implementing evidence-based practices and programs: *(1) exploration and adoption, (2) program installation, (3) initial implementation, (4) full operation, (5) innovation*, and *(6) sustainability.* Identifying these stages helps organizations and systems stay on track and aids in recognizing and solving common implementation problems in a timely and effective manner [6].

Emphasizing the importance of recognizing critical implementation steps, Fixsen et al. (2005) underscore the significance of understanding key factors that influence the success of upcoming resources in ECEC settings [6]. Their research and that of Grady et al. (2020) both indicate the presence of influencing factors at different levels affecting adoption and implementation [7]. These factors include staff behavioral components (knowledge, skills, beliefs, and attitudes), organizational aspects (compatibility, infrastructure, equipment, and leadership engagement) and external factors (financial and political components), all playing pivotal roles in the successful implementation of interventions [6, 7]. Furthermore, Grady et al. (2020) specifically mentions that the complexity, costs, adaptability, and the ability of digital interventions to meet user needs are essential for their adoption and implementation [7]. Based on Yoong et al. (2021), there are few studies that have explored barriers and facilitators related to the adoption and implementation of digital “healthy eating” interventions to improve ECEC food and nutrition environments [10].

In a prior study, we conducted a multi-component intervention within the context of Norwegian ECEC, addressing both environmental and individual-level determinants of healthy eating behaviors. Specifically, our digital “healthy eating” website intervention, Pre-schoolers’ *Food Courage 2.0* [11], tailored for 1-year-olds, successfully increased the consumption of targeted vegetables among children [11]. The staff were asked to carry out four components: (1) serve new lunch dishes 3 days per week, (2) introduce target vegetables during sensory playful sessions with the children, (3) follow health-promoting advice during mealtimes, and (4) collaborate with parents. A subsequent process evaluation revealed that ECEC teachers found the intervention to be educational and inspiring, despite encountering some challenges in cooking [12]. This feedback prompted adjustments to core components to facilitate broader implementation, benefiting diverse user groups and society. The adapted components were integrated into a new digital resource named *Nutrition Now*, designed to promote a healthy diet early in life across various settings [13].

The aim of this study is to identify challenges and supportive factors to optimize the adoption and implementation of a digital “healthy eating” resource in ECECs prior to implementation. *The research question guiding this study is: What key barriers and facilitators can influence the successful early-stage implementation of an upcoming digital “healthy eating” resource in ECEC settings, particularly from the perspective of teachers?*

## Methods

### Design

This qualitative study serves as a pre-study for the *Nutrition Now* project. It involved conducting individual interviews and focus groups with ECEC teachers (end-users). The main project, *Nutrition Now*, aims to implement digital interventions in both ECECs and healthcare centers within a Norwegian municipality [13]. This pre-study specifically focuses on the ECEC setting, and the results were used to guide the development of targeted strategies for optimal resource utilization prior to its rollout.

As an initial phase of *Nutrition Now* in spring/autumn 2020, qualitative interviews were conducted with ECEC teachers in the intervention municipality where the project was planned to be implemented. In the ECEC centers of this municipality, teachers had no prior experience with the digital resource, making it entirely new to them. The resource was not developed at the time and therefore not presented to them before or during the interviews. Our aim was for their feedback to reflect genuine needs, rather than opinions about a specific resource. The data from this study have been utilized to identify teachers’ perceptions and needs, and the results have been applied to enhance core components for our digital “healthy eating” resource in ECEC settings (unpublished findings, Helland, Bjorkkjar, Vejrup and Overby). Meanwhile, the results of the current study have been used to plan implementation strategies to enhance success in the pre-implementation stage.

The study adhered to ethical principles outlined in the Declaration of Helsinki. Approval was obtained from the Norwegian Centre for Research Data (26/03/2020, Reference 137,889) and our Faculty Ethical Committee (FEC). Reporting followed the Consolidated Criteria for Reporting Qualitative Research (COREQ) checklist to ensure comprehensive reporting.

### Setting and recruitment

All 59 public and private ECEC centers in the *Nutrition Now* intervention municipality were invited to participate in interviews during this project phase. The recruitment process involved reaching out to the ECEC network within the municipality. In January 2020, a seminar organized by the municipality informed all ECEC heads about the *Nutrition Now* project. Heads interested in participating responded to the municipal ECEC director. Fifteen ECEC heads consented to be contacted by the research team and received an email with study information and a consent form, which was then forwarded to relevant ECEC teachers.

Individual interviews occurred in two rounds: the first in May/June 2020 and the second in November 2020. All interviews, lasting 15 to 20 min, were conducted over the phone at times convenient for each ECEC teacher. Two focus groups were conducted via Zoom in early December 2020, with durations of 53 and 39 min. One of the authors (KV), unfamiliar with the participants, conducted all interviews and focus groups. Written informed consent was obtained from all participating teachers before conducting interviews and focus groups.

### Interviews

An information letter was dispatched to 15 participating ECECs, inviting teachers to engage in individual phone interviews as part of this study. Initially, eight ECEC teachers from seven private ECECs and one public ECEC volunteered to participate. Observing an uneven distribution between private and public ECECs, the researchers contacted the director of ECECs in the municipality. Following verbal invitations to the heads of municipal ECECs, four additional public ECECs consented. In total, 13 ECEC teachers were interviewed individually, representing 12 different ECECs. Notably, one interview involved two participants: a teacher and a head. Towards the end of each individual interview, participants were informed about their invitation to an upcoming focus group, providing an opportunity for further discussion on the findings.

### Group discussions

The data from the interviews underwent preliminary analysis before being presented to the focus groups. The results were organized according to the established core components of our existing “healthy eating” resource. These components cover: (1) the food and nutrition environment in ECEC, (2) educational activities involving food, (3) mealtimes, and (4) parental cooperation. Additionally, a new theme was introduced: government guidelines and documents. These five themes were included as subpoints under the second main topic in the focus group guide. The focus groups served as a complementary step following the individual interviews. The combination of individual interviews and focus groups aimed to deepen our understanding of participants’ perspectives and strengthen our initial findings by facilitating interaction and discussion among peers during group sessions. Information letters about focus group participation were sent to the heads of the 15 ECECs interested in the project, aiming to recruit between 9 and 12 teachers. Six teachers accepted the invitation. Due to the COVID-19 pandemic, the decision to conduct the focus group via Zoom led us to randomly divide the participants into two small groups, expecting this approach to encourage active participation and discussion. One registered teacher did not attend. The first group comprised two teachers from a private ECEC and one from a public ECEC, while the second group included two teachers from two different public ECECs. Four participants held the position of head teachers, one was an assistant head. Given the pandemic circumstances and the focus group’s purpose, we deemed the number of participants sufficient to gather valuable information beyond the individual interviews.

### Interview guides

Two semi-structured interview guides were employed, one for individual interviews (see Additional file 1) and one for focus groups (see Additional file 2). The individual interview guide covered ten topics: (i) Level of knowledge about early-life nutrition among staff, (ii) the current meal practice in the ECEC, (iii) thoughts about integration of activities focusing on children’s relationship with food in ECEC, (iv) thoughts about the use of a digital tool for accessing nutritional measure, (v) available cooking facilities in the ECEC center, (vi) involvement of children in cooking, (vii) opportunities for informing and training staff, (viii) ongoing pedagogical sessions and regular activities for children, (ix) barriers related to implementing a digital learning resource, and (x) challenges in implementing food and meal measure for children. This paper analyzes the following five topics: iii, iv, vi, vii, ix, and x.

The focus group guide, partially based on individual interview responses, covered four main topics: (i) general experiences in retrieving information from websites for ECECs, (ii) thoughts on the five core components in an upcoming digital “healthy eating” resource for ECECs based on preliminary interview results, (iii) considerations regarding the food and nutrition-related information needs of various user groups, including heads, teachers, food managers/chefs, staff in general, children, and parents. Lastly, (iv) discussions on the design of a “healthy eating” resource and any additional needs.

### Conducting interviews

The interviewer (KV) began by introducing herself and explaining the study’s objectives. Subsequently, she followed the respective interview guides. In the case of focus groups, participants were given a brief information video beforehand to prepare themselves, summarizing preliminary proposals for the design and content of the digital “healthy eating” resource from individual interviews. All interviews, both individual and focus group sessions, were audio recorded and transcribed verbatim by the interviewer and a master’s student in public health science. The questionnaire utilized in the interviews and group discussions conducted in this study has produced multiple findings, which will be published elsewhere (unpublished findings, Helland, Bjorkkjar, Vejrup, & Overby).

### Analysis

Thematic analysis was employed to analyze the qualitative data collected through interviews with ECEC teachers, following Braun and Clarke’s step-by-step approach [14, 15]. The aim was to systematically identify and code overarching themes. This involved independently coding transcribed data and coders met to discuss their perspectives on the data, which were then used to inform coding. The goal was to capture the most relevant aspects of the data, including themes less frequently discussed by teachers but deemed significant for implementing the “healthy eating” resource before its rollout. To ensure methodological rigor, two authors (KV and SHH) independently coded all transcribed data using a coding framework based on the research question and interview guide. This initial coding occurred in two stages: KV and a research assistant coded the data in NVivo, and later, SHH independently coded the data in NVivo. SHH meticulously read through all interviews, identifying noteworthy excerpts, grouping the text into meaningful units, and applying appropriate codes. These codes were then organized into main themes and subthemes. The evaluation and revision of themes were conducted to ensure clarity and differentiation between and within main themes and subthemes.

The same analytical process was applied to the focus groups, with SHH bringing an ECEC teacher and chef perspective, KV as a dietitian, and the research assistant as a public health nutritionist. SHH defined and named final themes, aligning them with the initial coding results from KV and the research assistant. The coders, including KV, SHH and NCO (a dietitian), gathered to discuss their interpretations of the data, which then influenced the coding process.

We took a deductive approach to our analysis, as we were specifically interested in identifying themes related to implementation. These themes were compiled into two figures, with illustrative quotations from the transcripts in running text. The authors, all women with Ph.D. degrees, employed previous research, relevant theory, and their insights to critically interpret the data for a comprehensive understanding of the findings. Interpretations were extensively discussed in consensus exchanges, recognizing the importance of reflexivity to enhance the study’s rigor.

## Results

### Participant characteristics

In this study, thirteen ECEC teachers engaged in individual interviews, comprising twelve women and one man. In one of the individual interviews, two teachers from the same center participated. All participants possessed a minimum of three years of higher education and held positions as teachers and/or heads in private or public ECECs within the same Norwegian municipality. The focus groups comprised four women and one man, all ECEC teachers or heads with similar educational backgrounds, representing three distinct public ECECs and one private ECEC. To distinguish individual ECECs in referencing quotes, each has been assigned a unique number within the range of 1 to 12, denoted as (I12), for instance. Focus groups are identified as FG.1 and FG.2 for clarity.

### Main findings

**Fig. 1:**
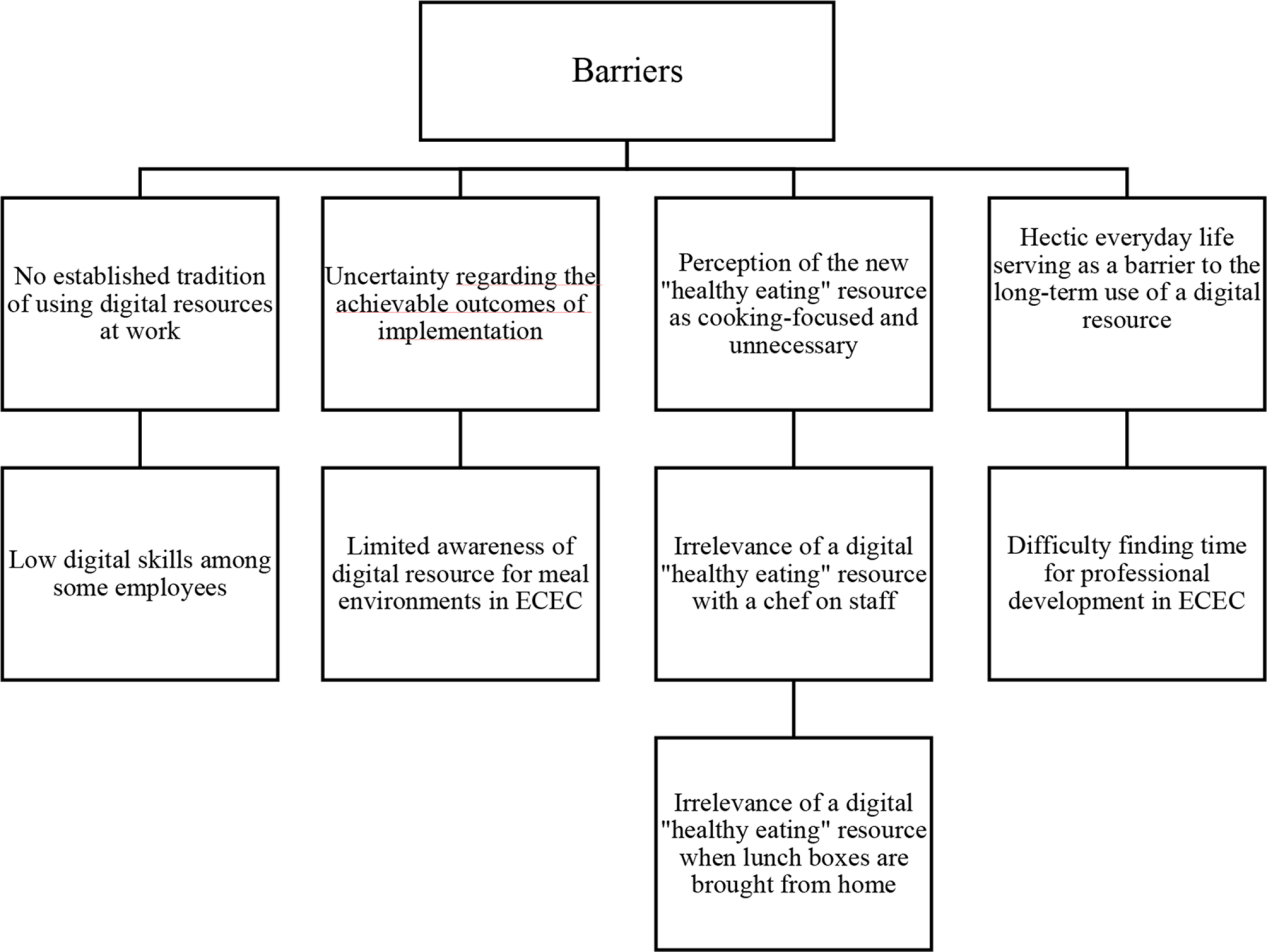
Identified implementation barriers

**Fig. 2:**
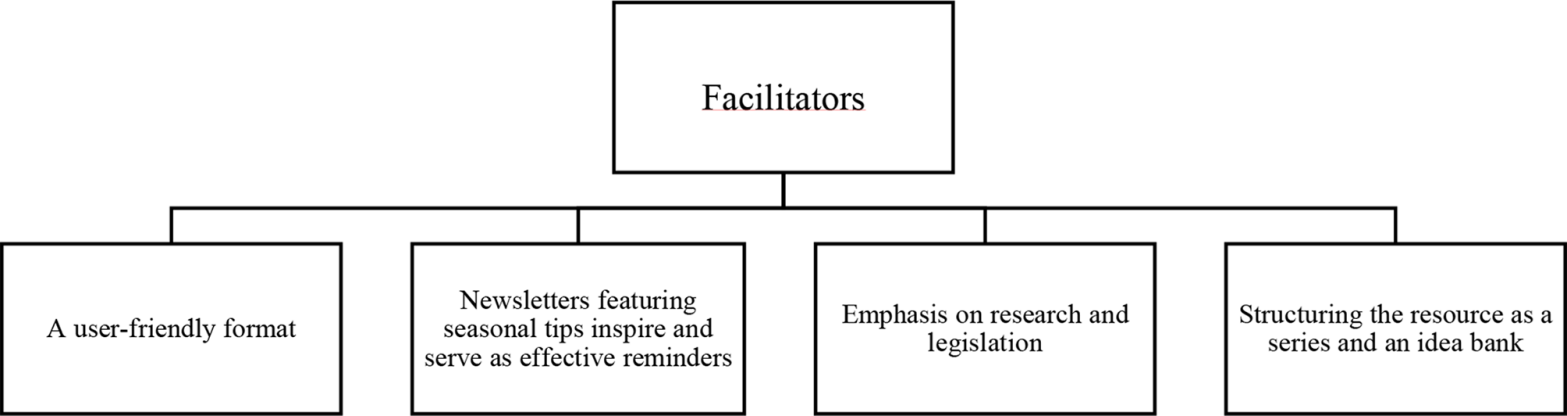
Identified implementation facilitators

Our study identified four key barriers (Fig. 1) and four supporting factors (Fig. 2) influencing the potential success of implementing the upcoming digital “healthy eating” resource in Norwegian ECEC. To offer a comprehensive insight, we delve into each theme, presenting relevant subthemes and illustrative quotes in the subsequent sections.

### Implementation barriers

Four main themes (Fig. 1) addressing potential obstacles related to the implementation of the upcoming digital “healthy eating” resource were identified.

#### No established tradition of using digital resources at work

The absence of a tradition of utilizing internet information at work poses a potential obstacle to the implementation of the upcoming digital “healthy eating” resource among the interviewed teachers. Their preference for traditional paper-based resources over incorporating websites into their work may hinder the integration (Fgr.2).

Despite some teachers acknowledge the availability of useful websites, they believe these resources are not fully exploited:

“We know that there are some good websites with materials for us to reflect on. It can be used with the children too, but it’s probably somewhat limited in terms of how much it is utilized.” (Fgr.1).

Furthermore, a teacher highlighted the existing competition among digital resources in their work context, expressing concern that introducing a new resource could add to this challenge. “We naturally want to have things of good quality, but many different websites on various topics keep emerging” (I1). The combination of a traditional use of tangible resources and perceived competition among digital resources can create resistance and overwhelm teachers, potentially serving as barriers to the effective implementation of the upcoming digital “healthy eating” resource in their work context.

#### Low digital skills among some employees

The potential challenges arising from varying levels of digital expertise among employees underscore the importance of ensuring the accessibility and simplicity of the digital “healthy eating” resource to facilitate its effective implementation. Three teachers highlighted concerns:

“Even today, when most people use digital tools, not everyone is equally comfortable with it. So, it has to be very accessible and simple; otherwise, it won’t be used correctly.” (I4).

“We have some older individuals who are hesitant about using technology and learning new things related to it. If there is an easy way to access it without too many steps, I think most people can manage it.” (I9).

“Not everyone is equally updated on it or fond of using it.” (I1).

#### Uncertainty regarding the achievable outcomes of implementation

One teacher expressed uncertainty about the potential benefits of implementing the upcoming digital resource for healthy eating, stating that they are not sure what could be achieved (I5). Another teacher couldn’t quite envision any benefits of such a resource, believing that teachers already have the skills to create activities on their own:

*“It’s a bit difficult to envision what the [benefits] should, um,. be in a way, other than … (pause) …, well we ECEC staff are really good at (laughter) coming up with ideas on our own…”* (I1).

#### Limited awareness of digital resource for meal environments in ECEC

The cultivation of healthy eating habits in children extends beyond providing nutritious food; it involves shaping social and physical environments during mealtimes. However, teachers seemed to overlook these aspects. In interviews, the focus leaned heavily toward recipes and food preparation, neglecting discussions about the social and physical environment surrounding mealtimes. This gap was particularly evident in a focus group where teachers were asked about their needs for support from the upcoming digital resource concerning the social environment during mealtimes. Rather than addressing these needs, one teacher solely emphasized recipe and cooking-related support.

Interviewer: “As I understand it, the mealtime settings are meant to be included there [in the resource] … how one eats, … physical and social frameworks … how you do it? … we need feedback from the users. …What do you need?”

Teacher: “Something that we know works, that takes into account that there are children and that it [dishes] are quite efficient to make… so that there are not many different operations to be performed…” (Fgr. 1).

Despite being briefed on the upcoming digital resource, which included tips about mealtime settings, some teachers expressed unfamiliarity with the term “frame of mealtimes.” One teacher even admitted uncertainty about its meaning (Fgr.1). Furthermore, one teacher seemed surprised that such a comprehensive topic as the social and physical environment in a mealtime setting would be included in a digital resource, expressing concerns about the complexity of the subject and its potential coverage (Fgr.1). The uncertainty regarding what can be achieved and the lack of knowledge or attention to the needs related to social and physical meal environments emerge as significant barriers to the implementation of the resource.

#### Perception of the new “healthy eating” resource as cooking-focused and unnecessary

Most teachers, including those interviewed (I2, I6, I10), associated the new digital resource with cooking-related concerns, particularly regarding time constraints, limited staffing resources, and low budget. Moreover, concerns were raised about the lack of cooking skills among staff:

“The challenges are the daily meals, that you need human resources and more competence. It’s crucial for me, I mean, it can always be someone who finds it nice to cook, but what competence do they have? … it should be proper food, …someone having expertise in this and cook, make purchases…”. (I10)

These concerns highlight potential obstacles to the successful implementation of the resource, emphasizing the need for an adequate number of staff and improved competence in cooking.

#### Irrelevance of a digital “healthy eating” resource with a chef on staff

In ECEC centers with an in-house chef, some teachers perceive a new digital “healthy eating” resource as less relevant. Delegating food and nutrition responsibilities to the chef diminishes the perceived necessity for such a digital tool. Insights from one teacher (I12), emphasizing the chef’s role in ensuring nutritious meals from which the rest of the staff learns, illuminate the dynamics within these centers. It appears that when a chef is present and teachers intuitively associate and limit a new digital resource on healthy eating to cooking, their need for such a resource seems to be minimized.

#### Irrelevance of a digital “healthy eating” resource when lunch boxes are brought from home

Two teachers (I11 and I12) noted that when children bring lunch boxes from home, the need for staff knowledge about diet and nutrition diminishes. In some ECECs, particularly those located in areas characterized by high socioeconomic status, where children bring lunches from home, the relevance of a digital resource on healthy eating may be deemed less significant. However, the importance of such a resource may vary in areas with differing socioeconomic statuses (I11). A teacher emphasized that in such cases, there might still be a need to educate teachers about the pedagogical opportunities of implementing a digital resource on healthy eating, especially in ECECs without food services (I1). This situation highlights the importance of reaching out to these establishments and ensuring they understand the pedagogical potential of such resources, even if they don’t provide meals.

#### Hectic everyday life serving as a barrier to the long-term use of a digital resource

The impending implementation of a digital “healthy eating” resource in Norwegian ECEC confronts challenges linked to teachers’ demanding daily routines. Teachers highlighted that the resource has the potential for short-term use, with the risk of being forgotten amid busy schedules (I12). One teacher observed a decline in the use of the ECEC’s own website for recipes (I3). Another teacher noted the excitement surrounding the resource but acknowledges that it may fade due to the multitude of platforms and the hectic nature of ECEC:

*“… It’s really fun… when you see something like wow, this was great fun, let’s use this. Yes, and then it falls back because there are many platforms, umm… Then it kind of slips away, and it’s somewhat easy to do because there’s so much happening in ECEC centers in general”.* (I1)

These challenges underscore the obstacles teachers face in establishing the long-term utilization of the upcoming digital resource in ECEC.

#### Difficulty allocating time for professional development in ECEC

Allocating time for professional development in ECEC poses a significant challenge due to teachers handling numerous responsibilities. Balancing these tasks makes it difficult to schedule additional meetings and training sessions, hindering the integration of new resources such as a digital “healthy eating” resource. Divergent opinions among teachers emerged regarding the feasibility of organizing informational meetings to introduce the digital resource. Teachers expressed concerns about the limited availability of time for information and training with few meetings often packed and challenging to allocate time for such activities, as noted by teachers I3, I5 and I9. Despite the lack of fixed staff meeting schedules, some teachers believed staff could familiarize themselves with the new digital resource during their planning time (I11).

In addition to formal training sessions, verbal reflections were highlighted as a valuable method for sharing experiences and insights among the staff. One teacher explained that professional development tends to manifest in actions: “We use a lot of verbal reflections, where we all sit and comment, and it works for us” (I8). This approach underscores the importance of ongoing dialogue and exchange of ideas among the staff members. In summary, the intricate balance of responsibilities, irregular staff meetings, and challenges in allocating dedicated time are aspects teachers face when implementing a new digital resource.

### Implementation facilitators

Several teachers expressed openness or interest in implementing a digital “healthy eating” resource, although they were uncertain about its appearance and practical application in daily ECEC work. When asked about the potential outcomes, one teacher emphasized the goal of increasing knowledge among staff, highlighting the importance of continuous learning (I8). Another teacher mentioned, “I would certainly have been curious about it, but I could not give a clear answer as to whether it was something I would use.” (I1). Four main themes (Fig. 2) were identified as facilitators for implementing a digital “healthy eating” resource to support teachers and other staff working in ECEC.

#### A user-friendly format

In the interviews, it emerged that for a digital resource to be effectively implemented, it needs to be user-friendly and easily accessible to all employees, including the chef. Participants in the focus groups emphasized the importance of a clear introduction page: underlining the need for a straightforward overview upon entering (Fgr.2). Additionally, two teachers from individual interviews suggested that ease of use and navigation are key factors in determining the resource’s frequency of use (I4). They recommended that the platform should be easily accessible, offering a wealth of practical ideas that can be readily implemented (I8). Teachers suggested that using an app, rather than a website, would be more convenient (Fgr.2). One teacher elaborated, noting that having the resource as an app on a phone or tablet allows them to have it readily available wherever they go (I12).

#### Newsletters featuring seasonal tips, inspire and serve as effective reminders

The teachers highlighted the importance of newsletters containing seasonal food-related tips as a crucial tool for implementing the upcoming digital resource. Emphasizing the importance of advocating for concise, impactful content with seasonal tips to inspire and remind. One teacher expressed:

“I think newsletters provide short but useful “bang” content, making you curious and keeping it relevant. With so many new things coming all the time, I can easily forget without a reminder… Now that it’s autumn, right? What ingredients are advisable now? Or for Christmas, referring to some recipes…”. (Fgr.2)

Regarding seasonal tips, teachers suggested integrating them into other pedagogical activities in ECEC to improve implementation (Fgr.1). One teacher expressed, “…link [it] to what’s happening in the ECEC center year. We are already connected to the season, and seasons and traditions, and yes… I think it could be a smart idea.” (Fgr.1).

A director at an ECEC noted a common issue where newsletters are filtered before reaching teachers: “A weakness with newsletters is that they often come to us leaders. Then it’s up to us what we do with them” (Fgr.1). However, they also mentioned that seasonal food-related tips could easily be linked to other seasonal pedagogical activities, increasing the likelihood of directly forwarding the newsletter to teachers. Overall, the teachers believed that utilizing newsletters with seasonal tips would effectively keep the resource in their minds and facilitate its implementation.

#### Emphasis on research and legislation

To facilitate the implementation of the digital “healthy eating” resource, teachers recommended *emphasizing the research and legislation*, considered essential for implementation. They emphasized the significance of promoting healthy eating habits in the ECEC and clearly communicating this message in the digital resource to encourage staff engagement:

“There are probably some who still think they can choose not to focus on it [nutrition] at all… …But you actually can’t. …because it’s in the curriculum. So, I think that could be an important entry point… … to get people on board.” (I1).

During focus groups, teachers stressed the importance of ensuring that all ECEC staff understand the rationale behind prioritizing healthy meals. They recommended communicating the societal mission of ECEC regarding the promotion of healthy eating habits (Fgr.1). They suggested featuring the term “research-based” in the resource and clearly stating the crucial role of a healthy diet for children (I1).

#### Structuring the resource as a series and an idea bank

The suggestions provided by teachers for crafting a digital “healthy eating” resource, such as structuring it as a series, allowing autonomy and freedom of choice, can be viewed as crucial implementation facilitators. One teacher recommended structuring the content as a series for continuity, stating:

“Perhaps there should be something that extends over a few times. …So that you can, in a way, maintain a thread in it… so that it doesn’t end up being like, wow, super fun, and then you visit the website, engage with it for a month or two, and then it somehow slips away.” (I1).

Additionally, several teachers expressed that autonomy and the freedom to choose content would be positive, one teacher mentioned; “…if you create a program … [where] others could kind of shop a bit, like an impulse or idea bank. That would be excellent, at least from my perspective” (I11). In summary, the teachers suggest that the resource should be designed with elements that are attractive, engaging, and flexible while providing teachers with autonomy and committing to long-term use. Identifying a design that aligns with these seemingly conflicting needs would facilitate the implementation.

## Discussion

This study explores the perspectives of teachers in Norwegian ECEC regarding the barriers and facilitators for implementing a planned and upcoming digital “healthy eating” resource, aiming to inform the selection of implementation strategies. While most teachers showed *interest* or positivity towards the new digital resource, a significant barrier exists in their *uncertainty* regarding the *achievable outcomes* of implementation. This challenge seems to arise from their limited perception of the resource, as they often associate it solely with *cooking* rather than recognizing its broader scope. Furthermore, several teachers lack experience integrating *digital resources* into their daily routines, anticipating challenges in *long-term use*. Allocating time for training and reflection sessions or *professional development in a hectic everyday life* was identified as a potential challenge. Key factors for promoting the adoption of the digital “healthy eating” resource include a *user-friendly* design and regular reminders through structuring parts of the content as *a series*, as well as receiving *newsletters* with seasonal tips regularly. Aligning the resource with *national policy and research* was suggested to enhance its feasibility and acceptance among ECEC staff. This study contributes to the understanding of relevant implementation strategies for improving healthy eating practices in ECECs by providing valuable insights into critical implementation steps and key factors, enriching the discourse on best implementation practices for such interventions.

In discussing our results illustrated in Figs. 1 and 2, we will structure the discussion under the following headings: (1) *barriers and facilitators during the “installation stage*” *of a digital resource*, and (2) *implications for the “initial implementation stage” of the digital resource*, guided by Fixsen et al. (2005). A summary of identified barriers and how they can be overcome in these early implementation stages is shown in Table 1.

**Table 1:** Summary of the identified barriers and how these barriers can be addressed in both the installation stage and initial implementation stage

Influencing factors	Barriers	Facilitators/ Ways to overcome barriers in the installation stage	Facilitators/Ways to overcome barriers in the initial implementation stage
Staff behavioral	No established tradition of using digital resources at work Low digital skills among some employees	Select staff members as champions Plan for training and anchoring Plan for continuous support, such as regular feedback, reminders, or incentives Design the resource in a user-friendly format Structuring the resource as a series and an idea bank Plan to send regular newsletters featuring seasonal tips to inspire and remind	Planning for leadership in the early stages, involves preparing champions to assist staff in changing practices and learning the core components
Organizational	Hectic everyday life serves as a barrier to the long-term use of a digital resource Difficulty finding time for professional development in ECEC	Stimulate the establishment of infrastructure and regular staff meetings to foster collective learning	
Political	Uncertainty regarding the achievable outcomes of implementation Limited awareness of digital resource for mealtime environments in ECEC Perception of the new “healthy eating” resource as cooking-focused and unnecessary	Promote clear communication about how the digital resource aligns with educational policy, research, and legislation regarding health-promoting practices related to food and mealtimes in ECEC settings	Plan for an early and clear understanding of all core components in the digital resource, and their relationship to governance documents
Financial	Perception of the new resource as cooking-related	Explore avenues for financial support to alleviate stress related to cooking	

Barriers and facilitators during the “installation stage” of a digital resource.

Our findings regarding the implementation of a digital “healthy eating” intervention during the installation stage will be discussed within the framework of four topics, each addressing unique challenges and required actions as outlined by Fixsen et al. (2005) [6].

Planning for training and continuous support.

Teachers’ ambivalence towards adopting a new digital “healthy eating” resource underscores the complexity surrounding implementation strategies in ECEC centers. While expressing both *interest* and *uncertainty* about its utility, our study contrasts findings from a previous Australian study indicating high intentions to adopt digital resources [16]. Although these studies are not entirely comparable in all aspects, various contextual factors may explain the difference. Moreover, earlier research indicates potential variation in engagement levels among ECEC centers when utilizing digital “healthy eating” resources [17], highlighting the need for further investigation into adoption factors and sustained use.

Teachers predicted *short-term* utilization, but they also believed that *newsletters with seasonal ideas* and *structuring the core elements of the digital resource as a series* would motivate use. This highlights the potential role of such strategies in supporting ongoing engagement. Prioritizing the training of teachers and staff, and building commitment, emerges as crucial steps during the program installation stage. Continuous support mechanisms, such as regular feedback, reminders, or incentives, are identified by several projects as essential for motivating and maintaining user engagement in the program [18, 19]. Given the barriers to the short-lived use of digital resources, the strategic selection of staff members as champions, responsible for the administration, training, and evaluation of practices, emerges as a promising strategy. This strategy is identified by Fixsen et al. (2005) and Barnes et al. (2021) as a central implementation strategy for fostering positive outcomes for stakeholders [6, 17], particularly in the context of children.

Planning for building organizational capacity.

Our study underscores a critical weakness in the culture of re*gular staff meetings*, which are vital for adopting new practices [6]. This finding emphasizes the importance of fostering an environment where teachers and staff can regularly convene to discuss and plan for the implementation of digital resources. However, it’s worth noting that the significance of regular staff meetings may vary across different countries and educational settings.

In line with previous studies, our study also identified *lack of time* and competing priorities as significant barriers to implementing digital interventions in ECEC settings [17]. To address these challenges, empowering and inspiring teachers and heads to prioritize the establishment of infrastructure and staff support is crucial [6]. These frameworks should facilitate planning, coaching, and evaluating staff teamwork [6]. Our findings underscore the importance of addressing *food and mealtime practices*, an area that received limited attention from the teachers. By prioritizing infrastructure and fostering a culture of regular staff meetings, ECEC organizations can enhance their capacity for successful implementation.

Highlighting digital resources as an integral part of educational policies and mandates.

Our study found that teachers emphasized the importance of aligning a new digital resource with the *national curriculum* for ECEC to generate interest. Additionally, they suggested *highlighting research* to promote understanding of the importance of offering children healthy and diverse food options in ECEC settings. By clearly incorporating how a digital resource aligns with educational policies and mandates, as described by Fixsen et al. (2005) and Damschroder & Hagedorn (2011), researchers contribute to anchoring the intervention [6, 20]. Highlighting this during the installation stage provides teachers with an early understanding of how the resource can be beneficial, thereby strengthening implementation [21].

Exploring opportunities for financial support.

In our study, when considering the implementation of a new “healthy eating” resource, teachers underscored barriers related to time and stress in *cooking*, in line with findings from our prior studies [12, 22]. To address this implementation issue, it’s crucial to secure funding [8], for example, at the state or municipality level. Implementing evidence-based interventions relies heavily on political and financial support [6, 21]. Therefore, prioritizing the securing of adequate funding and exploring alternative approaches to address the implementation challenges related to *cooking* in ECEC settings is crucial, preferably before the installation stage.

Implications for “the initial implementation stage” of a digital resource.

Planning for an early and clear understanding of all core components.

Based on our findings, researchers should carefully plan the introduction of digital “healthy eating” resources to prevent biases hindering adoption, particularly in the initial phase. Teachers may perceive the relevance of digital “healthy eating” resources as less significant when children bring *packed lunches from home* or when there is a *chef on staff*. Therefore, it is crucial to inform teachers or champions about the actual content and pedagogical possibilities, as outlined by Matwiejczyk et al. (2018) [1]. Mealtime in ECEC exists at the intersection of educational and health disciplines [23], necessitating the integration of health and education in introducing a “healthy eating” intervention. Our findings suggested that teachers paid less attention to *mealtime situations*. This highlights the need to explore effective implementation strategies to boost teacher engagement with this broad topic, which encompasses the establishment of eating habits and food literacy among children. Teachers’ perceptions of such resources may vary across countries, influenced by factors such as the food services system and the emphasis on food within the education system.

Planning for leadership and regular staff meetings in the early stages.

Our results indicated that staff have *limited time* to familiarize themselves with a new digital resource for ECEC. Implementers should carefully plan support for teachers or champions in the initial implementation stage. In this stage, staff are tasked with translating new core components into practice and have to acquire new skills and practices within their organizational context [24]. Anchoring the implementation of new digital resources with ECEC heads and ensuring alignment in goals between teachers and heads are recommended strategies, as suggested by Damschroder & Hagedorn (2011) [25]. Leadership is a key component for ensuring that “educational” staff understand the goals, distribute tasks, facilitate collaboration, and share experiences to develop practices, as noted by Blase et al. (2012) [26]. Additionally, engagement with digital intervention might be enhanced by encouraging champions to complete self-assessments and develop action plans [17]. The lack of allocated *meeting time* in ECEC could vary in other countries; however, clear leadership throughout the implementation process is essential in all countries.

While the most effective strategies for implementing guidelines, practices, or programs in ECEC settings are uncertain [8], ongoing Australian research investigates the potential of web-based implementation for support [2, 17, 27–29]. Our study underscores the importance of gathering data during the installation and initial implementation stages, as it provides valuable insight into the anchoring processes preceding full implementation. By utilizing data, such as iterative interviews conducted during implementation [30], we can gain a better understanding of the process. This approach can help inform the selection of strategies to enhance the introduction of digital “healthy eating” resources in ECEC settings. In summary, further research is needed to explore these strategies more thoroughly and improve our understanding of the implementation processes in ECEC.

Strengths and limitations.

This study used qualitative methods to explore possible mechanisms that can inform the selection of implementation strategies for digital “healthy eating” resources to support ECEC staff. The strengths of the study are outlined below. Qualitative interviews allowed us to comprehend the views of ECEC teachers and heads regarding the challenges and opportunities associated with implementing a new digital resource. Our study involved 13 teachers from both private and public ECEC settings, each with varying arrangements for food provision, contributing to a diverse range of perspectives. Moreover, our focus group included perspectives of five ECEC heads. The use of focus group facilitated the exchange and comparison of experiences among heads from four different ECEC centers, enriching the description of emerging themes from individual interviews. Furthermore, the small number of participants in each focus group (2–3 employees) ensured that each participant had ample opportunity to share their experiences, resulting in rich data. One of the authors who had not been involved in the development of the existing digital resource conducted all interviews, thereby minimizing the potential for bias in the findings. Additionally, two researchers with different backgrounds coded the interviews independently, and enhancing the rigor of the analysis. However, a limitation is that only one researcher (SHH) identified the final themes, these were critically reviewed by the other researcher (KV). The study also faces other limitations, including the small number of participants in the focus groups, which may compromise the generalizability and reliability of the findings. Additionally, the digital format of the study could potentially limit the diversity of perspectives and experiences captured in the interviews. Since the study was conducted in a Norwegian setting, caution is advised. Differences in public arrangements for food in ECEC and variations in national guidelines and curriculum may limit their transferability to other settings. Therefore, generalizing the findings of this study to other settings should be done with careful consideration of these differences. To gain a more comprehensive understanding of the unique barriers and facilitators for implementing digital resources in ECEC, future research should be conducted in different countries. This will allow for exploration of varying contextual factors and help identify implementation strategies that may influence the effectiveness of digital resources in promoting healthy eating habits in ECEC.

## Conclusion

This study revealed that ECEC teachers in a Norwegian municipality exhibited interest in exploring a new digital “healthy eating” resource, although there was an overall uncertainty regarding the extent to which it would be implemented. Nonetheless, our findings indicate that short-term engagement with the “healthy eating” resource is likely. Some teachers associated the upcoming “healthy eating” resource exclusively with the food environment in ECEC settings. This led to concerns regarding its suitability for implementation for various reasons. The teachers demonstrated limited awareness of the potential benefits of the digital “healthy eating” resource such as its ability to improve mealtime practices. The study advocates for several key strategies for enhancing successful implementation; prioritizing teacher training, offering continuous support, and identifying staff champions as pivotal components. Moreover, the significance of organizational capacity building, alignment with educational policies, and exploring financial support is emphasized. These insights contribute to the broader discourse on implementing digital resources in ECEC settings, offering valuable considerations for selecting implementation strategies that can ensure effective adoption and sustained impact.

### Electronic supplementary material

Below is the link to the electronic supplementary material.

Supplementary Material 1

Supplementary Material 2

## Data Availability

The data will be available to other researchers for non-commercial purposes upon request to the corresponding author.

## Abbreviations

ECEC: Early childhood and care
