# Supplementary material for: “I’m not sure whether I will implement it”: exploring barriers and facilitators to implementing a digital “healthy eating” resource in early education and care settings - teachers’ perspectives
Source: BMC Public Health. 2024 Jun 5;24:1499. doi: 10.1186/s12889-024-19014-7 (PMC11151519; doi:10.1186/s12889-024-19014-7)
Supplement: Supplementary file 1 — Supplementary Material 1 [file 12889_2024_19014_MOESM1_ESM.pdf]

Additional file 1: Interview guide - Individual interviews

Introduction: Presentation of the interviewer

Brief information: An e-learning resource is under development at the University of Agder for employees in Early Childhood Education and Care (ECEC) and healthcare centers. It aims to promote healthy eating habits among children by providing information and education to ECEC staff about children's food and meal habits. The resource includes measures to improve food quality, increase enjoyment of food, encourage children to try new foods, and create interactive meal experiences. These measures have been developed and tested at UiA with positive results.

Here's what we ask of you: We want employees to participate in interview to contribute to the exchange of experiences and information to develop the e-learning platform and adapt the measure so that it can be implemented in the everyday life of Norwegian ECEC.

Consent: Ask if the interviewee consents to being interviewed and to the interview being recorded. Inform that the recordings will be deleted after they have been transcribed.

Questions

i) **What do you think about the level of knowledge about early-life nutrition among staff?**
  - Is this a topic in education?
  - Do you think it's important to focus on this in ECEC settings?

ii) **Can you tell us how meal practice is currently conducted in the ECEC center?**
  - Number of meals?
  - Do the children bring packed lunches?
  - Do staff eat with the children?
  - How are the children seated at the table?

iii) **What do you think about integration of activities focusing on children's relationship with food in ECEC?**
  - Do you have any previous experience with this?
  - Have you participated in similar initiatives before?
  - Interest and motivation to participate in such activities in the ECEC?
  - What would it take to arouse interest in working on this?

iv) **What do you think about using a digital tool to access nutritional measure?**
  - Do you have access to PCs or tablets in the ECEC center?

v) **What cooking facilities do you have in the ECEC center?**
  - Cooking facilities and equipment

- Purchase of groceries
- Storage

vi) **What do you think about the involvement of children compared to what you do today?**

vii) What opportunities are there for informing and educating staff?

- Are there regular meeting days, educational session days, or planning days where education can take place?
- Would it be more attractive to undergo training if one could receive a certificate and it could be added to the CV?
- How do you think educating can best be conducted, introducing course leaders at gatherings with other ECEC staff or in groups within the ECEC with informational materials?

viii) Can you provide us with information about educational sessions and regular activities for children throughout the week, month, and year?

- Do you have a set annual schedule?
- Other relevant information about food and meals?

ix) What kinds of barriers can you imagine with the implementation of a digital learning resource?

x) What kinds of challenges can you imagine with the implementation of food and meal measures for children?
