# Supplementary material for: “I’m not sure whether I will implement it”: exploring barriers and facilitators to implementing a digital “healthy eating” resource in early education and care settings - teachers’ perspectives
Source: BMC Public Health. 2024 Jun 5;24:1499. doi: 10.1186/s12889-024-19014-7 (PMC11151519; doi:10.1186/s12889-024-19014-7)
Supplement: Supplementary file 2 — Supplementary Material 2 [file 12889_2024_19014_MOESM2_ESM.pdf]

Additional file 2: Focus group discussion guide

Introduction: Presentation of the moderator/interviewer

Brief information: We have invited you to discuss the needs that Early Childhood Education and Care (ECEC) facilities have for access to sources of information about food and meals. This project is a collaboration between your Municipality and the University of Agder with the aim of improving the diet of children in the municipality. The input you provide today will contribute to the development of an evidence-based website that will be offered to all ECEC facilities in the municipality and possibly at a national level in the future. We aim for it to become a resource for support and utility in everyday ECEC life. We are very pleased that **you could participate in this discussion.**

Instructions for the Focus Group Process: As you have been informed during registration, the discussion will take place via Zoom, and afterward, we will transcribe the conversation to analyze the inputs and assess how we can best create a website that is useful for ECEC. I just want to reiterate that the recordings will be deleted after they are transcribed, and all statements will be anonymized, so they are not linked to any individual person or ECEC center.

Before we begin:

- I kindly ask you to turn off your mobile phones.
- There are no wrong answers. Everyone has different experiences and perceptions of their situation, and that's exactly what we are interested in learning more about.
- My role will be to guide the conversation and keep us within the time frame of our one-hour meeting. I may interrupt at times and move on to the next topic to ensure we cover all the themes.

Questions

Introduction: Quick round of introductions, including your names, the ECEC center you represent, and approximately how many children attend the ECEC.

i) **General experiences in retrieving information from websites for ECEC centers, not necessarily specifically related to food and mealtimes**

ii) **We've developed a proposed structure for the website content, based on mealtime routines and educational activities in ECEC everyday life. We've divided these into five main categories:**
   - Food served - breakfast, lunch, afternoon snack, snack, outdoor meals, cold and hot food
   - Mealtime frameworks – physical and social frameworks
   - Educational activities involving food, apart from mealtimes
   - Governing documents
   - Parent/home collaboration

Show slides of the five main categories and discuss the structure.

- What do you think about a structure that is based on these categories?

iii) We want to explore these categories based on the different user groups and the type of information they may need; Exploring from various perspectives: Manager, Teacher, Food manager (chef), ECEC Assistants, Children, Parents.

- From the perspective of different groups?
- Are there any user groups you believe are missing?

iv) Discussions on the design of a "healthy eating" resource and any additional needs

- Design?
- Key elements?
- Newsletters?
- Recurring activities/theme of the month?
- Blog/discussion forum for ECEC centers?

Closing question: Is there any topic we haven't covered that you'd like to bring up?

Note: Topics Interviewer should be aware of during the discussion: Challenges in implementing activities.
